# Progressive and Asymmetrical Deadlift Loads Captured by Wearable Motion Tape Sensors

**DOI:** 10.3390/s24237700

**Published:** 2024-12-02

**Authors:** Elijah Wyckoff, David Sten, Regan Wareham, Kenneth J. Loh

**Affiliations:** 1 Active, Responsive, Multifunctional, and Ordered-Materials Research (ARMOR) Laboratory, Department of Structural Engineering, University of California San Diego, La Jolla, CA 92093, USA; ewyckoff@ucsd.edu; 2 S10 Fitness, San Diego, CA 92110, USA; dave@s10fitness.com (D.S.); reganwareham@gmail.com (R.W.)

**Keywords:** deadlift, training, human performance, muscle, nanocomposite, posture, skin, strain, wearable sensors, wireless electromyography (EMG), training

## Abstract

Weight training is widely adopted and highly effective for enhancing both muscular strength and endurance. A popular weightlifting exercise is the deadlift, which targets multiple muscle groups including the lower back, glutes, and hamstrings. However, incorrect technique (i.e., poor form) can slow training progress, result in asymmetrical muscle development, and cause serious injuries. The objective of this study was to validate that a self-adhesive, elastic fabric, wearable, skin-strain sensor called Motion Tape (MT) could monitor a person’s posture while performing deadlift exercises. Two pairs of Motion Tape were attached on the front and back sides of the pelvis at each posterior superior iliac spine to record muscle engagement during deadlift exercises. The results of this preliminary study confirmed that the MT identified asymmetry in muscle engagement during deadlifting repetitions. In addition, the sensors could quantify the different levels of effort exerted according to the deadlift weight load.

## 1. Introduction

One main goal of weightlifting is to build strength by progressively challenging the muscles with increasing resistance. Deadlifting is a weightlifting exercise that involves lifting a loaded barbell or bar from the ground to the level of the hips, before lowering it back down to the ground. However, improper form and technique can lead to asymmetrical muscle development, hamper training progress, and cause painful acute and chronic injuries. The conventional pathway for strength training, particularly for novices, is to be instructed by a physical trainer who can help assess posture and correct movement. More advanced weightlifters may opt for reviewing self-recorded videos. However, both these methods are highly subjective, creating the need for an objective measurement solution.

Augmenting training with objective measurements allows for form analysis and can help prevent injuries resulting from improper technique. For example, noncontact computer vision-based systems, such as optical motion capture (mocap) using high-resolution cameras and advanced algorithms, have been demonstrated to measure deadlift form and to track body movements [1,2]. New advances, such as the Microsoft Kinect, have also been shown to successfully facilitate real-time monitoring [3]. Although these systems can accurately measure body kinematics, the ability of the human body to compensate for weak musculature in certain regions suggests that both posture and muscle engagement measurements are needed to understand how functional movements are executed.

In recent years, wearable sensors have revolutionized personal fitness and health monitoring by providing a cost-effective and portable solution for acquiring real-time data during various physical activities. Inertial Measurement Unit (IMU) sensors have been attached to a barbell to measure displacement and to estimate the amplitude of each deadlift repetition [4]. This technique accurately measures the result of the movement but lacks information on how the body executes the deadlift. On the other hand, surface electromyography (sEMG) sensors detect the electrical potential generated by muscle fibers and have been used during deadlifting to measure lower back, gluteal, and hamstring muscle engagement [2,5]. Providing information on muscle engagement is critical for form analysis, but sEMG sensors are not designed to be worn by athletes because of their form factor and issues with movement artifacts. Integrating both IMUs and sEMG via a body sensor network enables the monitoring of joint angles and muscle activity in three dimensions [6]. Another wearable solution with a network of sensors is a pressure sensor suit based on flexible piezoresistive materials, which detects muscle strain and monitors posture in real time [7]. These methods are effective in analyzing deadlifting form based on body kinematics but require systems of many sensors. This reliance on bulky multi-sensor setups introduces discomfort that can adversely affect weightlifting form, which highlights the need for an integrated user-friendly solution.

While mocap and wearables such as sEMG provide accurate insights into movement and muscle activity, their upfront investment, form factor, and limited accessibility prevent their widespread use, particularly among amateur weightlifters. Thus, the aim of this study was to validate that a self-adhesive, elastic fabric, wearable, low-cost, skin-strain sensor, called Motion Tape (MT) [8,9,10], could monitor differences in muscle engagement during progressive deadlift loads while also identifying any asymmetry in the weightlifter’s form. One amateur weightlifter served as the participant for this study, and Motion Tapes were fabricated and worn by the participant while performing deadlifts. Standard deadlifts with different weights were performed, and external factors were also introduced to purposely induce and reduce asymmetry. Motion Tape measurements were compared with sEMG sensor measurements for validation. While the designed experiment involves testing and measurement issues, these processes are necessary to derive actionable insights for both trainers and weightlifters.

## 2. Materials and Methods

### 2.1. Motion Tape Fabrication

**Figure 1:**
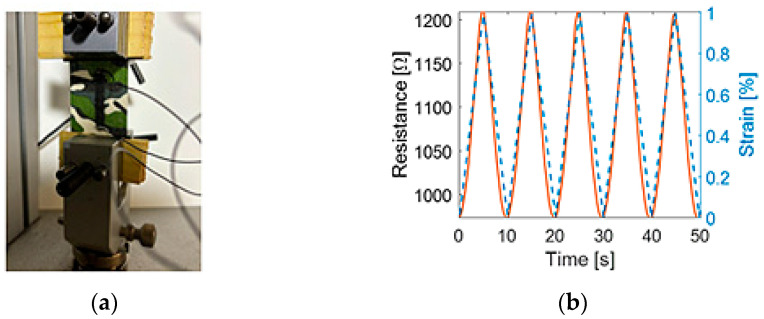
The sensor characterization test entailed straining (**a**) MT in the longitudinal direction, and (**b**) the MT resistance time history is overlaid with the applied tensile cyclic strains.

Lin et al. [10] reported the fabrication procedure for spray-coating a dispersed graphene nanosheet and ethyl cellulose solution onto commercial kinesiology tape (K-tape) substrates. For this work, a 2% multi-walled carbon nanotube (MWCNT) aqueous solution was employed (Figure 1) [11]. It was shown that, when MWCNT films are strained, the conformation of the nanocomposite changes, thereby leading to a measurable change in the bulk electrical resistance [12,13]. This linear relationship is demonstrated by the load frame electromechanical test, and the corresponding results are presented in Figure 1. K-tape (Rock Tape, Durham, NC, USA) substrates were prepped by applying ethanol to enhance fabric hydrophilicity. The MWCNT solution was then drop-casted onto a 4 × 0.75 cm^2^ masked area, for a total of three times, to form a piezoresistive nanocomposite with a baseline resistance (*R*_0_) of ~1 kΩ. Once the film dried, conductive silver paste (from Ted Pella, Redding, CA, USA) was applied to both ends, and multi-strand wires (from Digi-Key, Thief River Falls, MN, USA) were soldered for the ease of electrical resistance measurements [14].

### 2.2. Wireless Sensing Node

A wireless sensing node was custom designed and prototyped to collect MT data at a sampling rate of ~65 Hz. The printed circuit board (PCB) included a Texas Instruments (Dallas, TX, USA) CC1350 micro-controller unit and transmitted the measured signals via Bluetooth to a laptop computer. Data were recorded using SmartRF Studio 7.2. Once all MT resistance data were stored and imported in Matlab R2023a, a Hampel filter was applied to remove outliers [15]. The main focus of this study was not on the wireless sensing node itself, and more details regarding its design and development are reported in Pierce et al. [16].

### 2.3. Human Participant Deadlift Study

This human subject study was approved by the University of California San Diego, Institutional Review Boards, Human Research Protection Program, under Project No. 191806X. Informed written consent was obtained from the participant, who self-reported as an intermediate level weightlifter and collegiate athlete. This study focused on one participant, as opposed to multiple participants, because the aim was to investigate how perturbations applied during deadlifting would affect an individual’s muscle and movement responses, as captured by the sensors employed. Differences in biomechanics and how people move would suggest that these results would not be translatable across different participants. Instead, multiple tests were conducted with the same participant to ensure the statistical significance of any differences observed.

**Figure 2:**
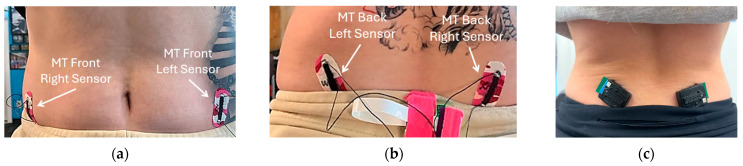
Position for MT placement on the (**a**) front and (**b**) back of the participant, with (**c**) sEMG placement on the back of the participant.

Two pairs of Motion Tape were attached, at an ~60° angle with respect to the waist, on the front and back sides of the pelvis to match the angle of each posterior superior iliac spine (PSIS) and anterior superior iliac spine (ASIS) (Figure 2a,b). When affixed above the PSIS and ASIS, it was hypothesized that the Motion Tape could detect subtle deformations in the skin caused by muscle expansion and contraction in the glutes and latissimus dorsi on each side. A previous study by Lin et al. [9] found that an increase in Motion Tape electrical resistance corresponded to tension, while a decrease was due to compression. Furthermore, the study also showed that the electrical resistance amplitude of the Motion Tape, when placed on the biceps and forearms, was correlated to the sEMG measurements and the weight of the dumbbell lifted during biceps curls [14]. Thus, this study tested the hypothesis that the MT could measure changes in resistance (i.e., due to variations in skin strains) on the lower back region in response to different degrees of muscle engagement. This provides significant information on how these muscles facilitate shoulder and hip extension in gait and during deadlift. This applied understanding of symmetry and amplitude of muscle engagement can guide trainers and weightlifters aiming to optimize functional movements during strength-based exercises. In addition, sEMG measurements were acquired in parallel and at the same locations to compare the MT to latissimus dorsi muscle activity (Figure 2c). The Delsys (Natick, MA, USA) Trigno Avanti sEMGs were used. Gait assessment was also performed before deadlifting began and in between deadlift sets to qualitatively assess the deadlift performance.

**Figure 3:**
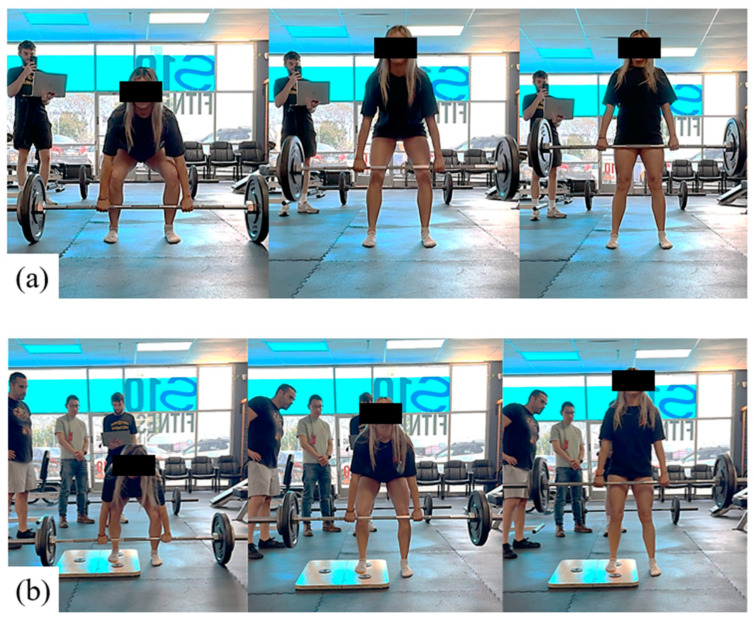
(**a**) Standard deadlifts were performed (Sets #1 to #3), and (**b**) external factors, such as an unbalanced platform under right foot (Set #4), were also introduced during deadlifting.

The six different deadlift sets performed by the participant in this study are summarized in Table 1. Five repetitions were performed during each set, and the weights were selected to target a maximum self-reported rate of perceived exertion (RPE) of 5. The RPE is a subjective score from 0 (no exertion) to 10 (maximum exertion). This value was selected to safely study the effects of external influences for the final three sets of deadlifts, with Set #4 intended to purposefully induce asymmetry by placing a platform under the subject’s foot on their dominant side (Figure 3). Set #5 was designed to reduce asymmetry observed in the subject by unilaterally loading one side of the barbell. Set #6 was also designed to reduce asymmetry by attaching an elastic band to one side of the barbell to force the subject to stabilize bilateral muscle engagement. The participant performed each deadlift set in the order shown in Table 1, before repeating the entire sequence following adequate breaks.

For all the performed sets listed in Table 1, clear Motion Tape tension readings recorded by the front sensors indicate that lumbo-pelvic stability was maintained throughout the deadlift repetition, where the hips were extended to induce peak strain at the top of the repetition. On the other hand, readings from the back MTs did not produce a distinguishable difference from set to set, despite the indication of muscle activation from the sEMG datasets. Therefore, it was determined that only data from the two front MT sensors would be used for this initial analysis to begin understanding the influence of muscle engagement on the kinematic measures from the MT.

### 2.4. Data Processing Methods

Motion Tape electrical resistance data streams were collected using the wireless DAQ node during the tests. The normalized resistance change (*R_n_*) was calculated by

(1) $$R_{n}=\frac{R-R_{0}}{R_{0}}$$

*R*_0_ corresponds to the baseline resistance of Motion Tape after it was affixed to the participant and when the participant was in a relaxed, standing, and neutral position. *R* is the electrical resistance of MT at any given instance during testing. Here, *R_n_* was processed using a method of locally weighted scatter plot smoothing, or Lowess, to reduce noise.

Muscle engagement measurements were also acquired during Sets #1 to #3 (Table 1). Aside from recording the raw sEMG waveforms for the left and right muscle groups, the sEMG envelope was derived by root mean square (RMS) sliding, where N is a window size of 100 ms:

(2) $$RMS(t)=\sqrt{\frac{1}{N}\sum_{i=t}^{t+N-1}{sEMG(i)}^{2}}$$

From this, muscle engagement was quantified during Sets #1 to #3 to verify differences in muscle activation as the participant performed heavier deadlifts from 95 lb to 135 lb.

To assess the degree of asymmetry in the participant’s posture during deadlifting, the mean absolute error (MAE) was calculated for each set. The MAE is a quantitative assessment of consistency and symmetry in MT strain measurements between the left and right sides, which was calculated as a ratio:

(3) $$MAE=\frac{R_{L,peak-avg}}{R_{R,peak-avg}}$$

For each deadlift set, the peak MT *R_n_* values during each repetition were identified. Then, the average of all the peak values of different sets was computed for each of the left (*R_L,peak-avg_*) and right (*R_R,peak-avg_*) sensors. Furthermore, using Equation (3), a similar calculation can be used to find the MAE with the peak sEMG signals.

## 3. Results and Discussion

### 3.1. Standard Deadlift Sets #1 to #3

**Figure 4:**
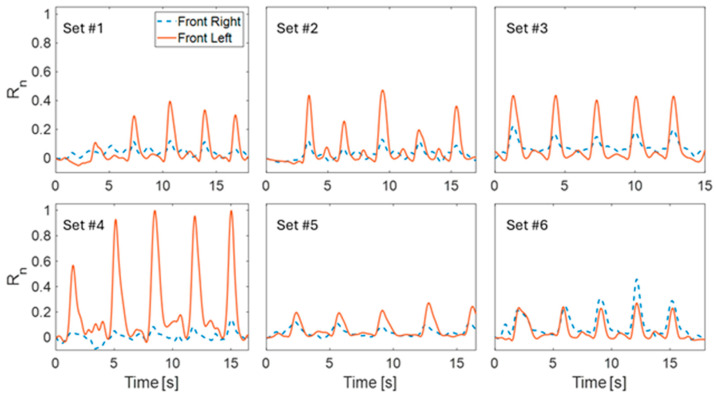
Normalized plots of resistance data for all sets of deadlifting numbered according to set number.

The Motion Tape results, sEMG measurements, and MAE calculations for the standard deadlift sets (i.e., Sets #1 to #3) were first analyzed. *R_n_* plots of the measured skin-strains for the front left and right MTs for a sequence of six deadlift sets are presented in Figure 4. The plots in Figure 4 for the first three sets (Sets #1 to #3) show, on average, an increasing MT response correlated to the progressive weight increase. These results are even more obvious in Table 2, where each Motion Tape showed an increase in average *R_L,peak-avg_* and *R_R,peak-avg_* as heavier weights were lifted from Set #1 to Set #3. Although *R_L,peak-avg_* and *R_R,peak-avg_* (in Table 2) on its own may not provide strong physical meaning, their value comes from comparing the Motion Tape response among different sets or between the sensors, as was performed in another study focused on the lower back region [17]. To evaluate the relationship between the increase in weight and average peak values, a Pearson correlation analysis was performed. The analysis showed a positive correlation for both the left MT (*r* = 0.905) and right MT (*r* = 0.999).

**Figure 5:**
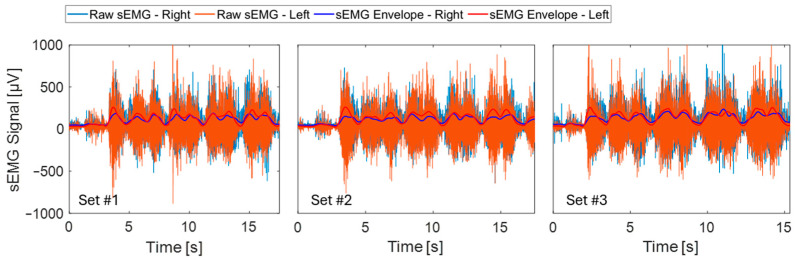
The sEMG raw data for the left and right muscle groups captured during Sets #1 to #3 were also overlaid with the corresponding sEMG envelope.

Figure 5 plots the raw sEMG for the left and right muscle groups, which is also overlaid with the sEMG envelope. The sEMG average peak values are reported in Table 3. Similar to the Motion Tape, an increasing trend of sEMG average peak values as heavier weights were lifted was observed, matching the results for the Motion Tape; however, the sEMG results for Sets #1 and #2 were approximately the same. Correlation coefficients between the average peak values from Table 2 and Table 3 for MT versus sEMG were found to be stronger for the right side (*r* = 0.832) than the left (*r* = 0.560). This mild correlation is expected, because the Motion Tape measures skin strains derived from both movement and changes in muscle engagement [8,9,10]. In contrast, sEMGs capture strictly muscle engagement, with potential errors introduced from movement artifacts such as skin strains. In general, the results confirm that greater muscle engagement was observed as the participant lifted heavier weights, and this phenomenon was also apparent from the Motion Tape measurements.

It was observed during the study that the participant, who is right-hand dominant, compensated for weakness on the left by exerting greater effort to pull more with the left side. This can also be observed from the MT data in Sets #1 to #3 in Figure 4, where the front left sensor showed a significantly greater amplitude in *R_n_* versus the right. The MT contralateral to the subject’s dominant side consistently showed higher strain values compared to the ipsilateral sensor, indicating that the subject’s left side was likely compensating more during the lifts and perhaps also experiencing greater movement. It was also observed in Figure 4 that the peak strain for the left MT tended to marginally lag the right. This provides more evidence that the subject led with the right side, requiring more effort on the left, which was further confirmed by higher values recorded on the left muscle group with sEMG.

Table 2 also confirmed that the average peak *R_n_* was greater for the left versus the right MT. Consequently, the Motion Tape MAE values (as calculated using Equation (3)) for each set were all greater than 1, suggesting asymmetry towards the left. It should be emphasized that this phenomenon was a result of greater strains induced on the left side of the body, rather than differences in the MT sensitivity, because all sensors were fabricated simultaneously in the same batch. Pearson correlation analysis was also performed on the MAE values from the MT and sEMG, resulting in a strong positive coefficient (*r* = 0.997), which validated the results obtained by the MT with regard to asymmetrical muscle engagement. Detecting asymmetry informs trainers and promotes the application of corrective measures for the weightlifter, and the effectiveness of these interventions can be objectively evaluated through subsequent MT measurements. Ideally, this would demonstrate a reduction in asymmetry, which was the aim of Sets #5 and #6, as discussed in the next section.

### 3.2. Perturbed Deadlift Sets #4 to #6

The perturbed deadlift sets were designed to force changes in the subject’s posture during deadlifting. First, Set #5 was designed to induce higher levels of strains on the left side due to the introduction of the unbalanced platform. Set #4 in Table 2 showed that *R_L,peak-avg_* is 0.889 and is larger than *R_R,peak-avg_*, which is 0.073. It should be noted that the significant increase in *R_L,peak-avg_* was not just the result of greater muscle engagement but also included the effects of greater movement from pulling the weight a farther distance.

Second, Sets #5 and #6 were designed to correct the subject’s intrinsic asymmetry. The corresponding Motion Tape results in Figure 4 demonstrate that the subject was able to achieve better balance in muscle engagement with both the uneven weight and band pull. The perturbation intended to balance the subject (as a means of form correction) was also quantified by the lowest MAE readings of 2.31 and 0.814 (in Table 2) for the unilateral loaded weight (Set #5) and the forward band pull (Set #6), respectively.

### 3.3. Other Considerations

In this study, the participant wore the same set of Motion Tape and sEMG sensors throughout the entire test. This was intentionally planned to ensure that the sensor results were comparable between different tests and sets. However, it is known that any slight change in sEMG position could result in a drastically different muscle engagement measurement [18]. Similarly, the MT response can also be affected by its placement. If new Motion Tape was applied and positioned at a slightly different location or angle with respect to the previous MT sensors, the expectation is that the new sensor readings would be slightly affected due to a position error. These differences may be minor, since the Motion Tape measures the average skin strains below its sensing region, which is defined by the size of the nanocomposite sensing element deposited [10]. However, future work is required to understand how placement errors affect MT sensing streams, which is beyond the scope of this current study. It should also be mentioned that the results presented in this work are subject-specific and depend on how the participant performed the deadlifts.

## 4. Conclusions

The objective of this work was to validate that Motion Tape could monitor differences in posture, movement, and muscle engagement during deadlifting to provide form feedback for enhanced sports training. The test results indicated that Motion Tape is an effective tool for monitoring muscle engagement in progressive deadlifting loads while identifying asymmetry in form. Two sensors worn on the front of one subject successfully detected strain differences, revealing significant asymmetries in kinematic patterns on either side of the body caused by asymmetrical muscle engagement, which were also observed by the sEMG data. This was particularly evident during the initial sets and when an unbalanced condition was induced, demonstrating the Motion Tape’s sensitivity to variations in lifting form. Corrective measures were shown to reduce asymmetry, which was confirmed by the Motion Tape measurements. The results from the front sensors indicate that the MT could not only identify imbalances in lifting form kinematics but could also assess the efficacy of corrective strategies, providing valuable feedback for optimizing the lifting technique. This study demonstrated that MT is a valuable noninvasive solution for the real-time monitoring and analysis of weightlifting form and can offer detailed insights for both trainers and weightlifters based on muscle engagement and symmetry that will be further explored in future works. Such insights could help athletes augment their movements and behavior for the purposes of increasing overall performance.

## Figures and Tables

**Table 1:** Summary of experimental deadlift sets.

Set Number	Weight	RPE	Description
1	95 lb/43.1 kg	3	Normal deadlift
2	115 lb/52.2 kg	4	Normal deadlift
3	135 lb/61.2 kg	5	Normal deadlift
4	135 lb/61.2 kg	5	Unbalanced, platform under right foot
5	145 lb/65.8 kg	5	Unilateral load (weight added to left side of barbell)
6	135 lb/61.2 kg	5	Unilateral band pull (left side of barbell)

**Table 2:** Motion Tape summary of results.

Set Number	Average Peak Value		MAE, Left vs. Right $(\left|Ω/Ω\right|$)
	Front Left *R_L,peak-avg_*	Front Right *R_R,peak-avg_*	
1	0.287	0.093	2.89
2	0.435	0.141	3.10
3	0.450	0.188	2.39
4	0.889	0.073	12.2
5	0.231	0.099	2.31
6	0.237	0.300	0.814

**Table 3:** Results from sEMG experimental analysis.

Set Number	Average Peak Value		MAE, Left vs. Right $\left(\mu V/\mu V\right)$
	Left [μV]	Right [μV]	
1	229.1	158.7	1.44
2	228.8	157.1	1.46
3	248.8	181.43	1.37

## Data Availability

The data presented in this study are available upon request from the corresponding author. The data are not publicly available, owing to ethical concerns, as they were obtained in a clinical trial.
